# Developmental Links Between Children’s Working Memory and their Social Relations with Teachers and Peers in the Early School Years

**DOI:** 10.1007/s10802-015-0053-4

**Published:** 2015-07-30

**Authors:** Amber de Wilde, Hans M. Koot, Pol A. C. van Lier

**Affiliations:** Department of Developmental Psychology and EMGO Institute for Health and Care Research, VU University, Van der Boechorststraat 1, 1081BT Amsterdam, The Netherlands

**Keywords:** Working memory development, Teacher-child relationships, Peer relationships

## Abstract

This study assessed the developmental links between children’s working memory development and their relations with teachers and peers across 2 years of kindergarten and early elementary school. Kindergarten and first grade children, *N* = 1109, 50 % boys, were followed across 2 school-years. Children were assessed across 3 waves, in the fall and spring of the first school-year (within school-year), and finally in the spring of the second school-year. Working memory was assessed using a visuo-spatial working memory task. The developmental links between working memory and child-reported teacher-child relationship quality (warmth and conflict) and peer-nominated likeability and friendedness were assessed using autoregressive cross-lagged models. Lower working memory scores were related to increases in teacher-child conflict and decreases in teacher-child warmth one school-year later, in addition to decreases in likeability by peers within the same school-year. Conversely, teacher-child conflict was negatively associated with the development of working memory across the studied period. Path estimates between working memory and social relational factors were similar for boys and girls. Findings show developmental links between working memory and social-relational factors and vice versa. These results suggest that children’s working memory development can be fostered through pro-social relations with teachers in early elementary school children.

Working memory is the cognitive process of keeping a limited amount of information in the focus of attention and manipulating it during a short period of time (Klingberg et al. 2002a, b). It is one of the executive function skills that are essential for organizing, executing, and inhibiting behavior, and hence are indispensable for children’s functioning in social situations (Riggs et al. 2006). Higher working memory capacities have been reported to facilitate the social development of children (McEvoy et al. 1993; Riggs et al. 2006). Conversely, the development of working memory itself may be affected by children’s social experiences (Riggs et al. 2006). Indeed, although generally seen as a trait, working memory has proven malleable, particularly in younger children (Klingberg et al. 2005; Klingberg et al. 2002a, 2002b), and develops until young adulthood (Gathercole et al. 2004; Riggs et al. 2006). Positive social experiences have been suggested to foster, while negative social experiences may deplete children’s cognitive development (Baumeister et al. 2002, 2005; Davies et al. 2008; Hinson et al. 2003; Thijs and Koomen 2008). Despite this plausibility, research on the reciprocal developmental links between children’s school-based social experiences with teachers and peers and working memory is, to our knowledge, lacking. The objective of this study is therefore to test cross-time reciprocal links between children’s social experiences with teachers and peers, and their working memory across 2 years in kindergarten/ early elementary school.

## Developmental Links Between Working Memory and Social Experiences

Working memory is an important factor in children’s social development as the ability to process social information is thought to be dependent on working memory. Adequate processing of social information is essential for the development of children’s social cognition and behavior. Indeed, lower working memory capacities have been thought to leave children less equipped to deal with, and behave appropriately in, (novel) social situations (Monks et al. 2005; Shallice et al. 1996). This may especially signal a problem for children transitioning to kindergarten and elementary school. With this transition, their social world widens drastically with the emerging relations with teachers and age-matched peers in which children have to function for a significant portion of time of the week. Indeed, although many children in western societies attend daycare before entering kindergarten or primary school, daycare is different from formal schooling in that the adult-child ratio in daycare is generally higher and formal schooling comes with increased performance standards, both academically and socially (Rimm-Kaufman and Pianta 2000). In line with this, studies showed that low working memory performance was associated with peer rejection (McQuade et al. 2013), and that among children with attention and behavior problems, executive functions, including working memory, were related to social problems in school (Fahie and Symons 2003).

It is important to note that the cited studies above hypothesized an influence of poor working memory on children’s social outcomes. There is however emerging theoretical and empirical evidence suggesting that in additionally to such links, reverse paths, in which social experiences affect working memory development may exist. According to the regulatory depletion model, stress in the regulation of one domain (i.e., the social domain) results in a depletion of a shared pool of resources, thereby causing impairments in other domains (i.e., working memory or executive functioning in general) (Davies et al. 2008). Stressful social environments, for example those characterized by conflictual social relations with teachers or peers, demand more of children’s working memory than would be required in positive everyday social interactions (Monks et al. 2005; Shallice et al. 1996). That is, the stressful social encounters may make children feel less safe and secure within the school. The attempts of children to mitigate this stress by restoring their troublesome social relations, will require children’s energy, thereby depleting children’s bio-psychological resources (Davies et al. 2008; Williams 2009). Moreover, if restoring relations does not turn out successful, Williams (2009) argues that the next, final phase is to give up, further depleting children from opportunities to practice working memory. Similarly, according to work by Baumeister and colleagues, humans are programmed to form stable, positive interpersonal relationships, which facilitate children’s learning and cognitive development (Baumeister et al. 1996, 2002, 2005). According to this, not having such positive interpersonal relationships may lead into a momentary state of cognitive impairment or deconstruction in which someone shows impairment in responding to tasks that require cognitive functioning (see also Williams 2007). As non-positive social experiences in kindergarten and elementary school tend to be stable (Howes and Hamilton 1992; Howes et al. 1998; Ladd 2006; Sturaro et al. 2011) it seems likely that these have an impact on children’s cognitive development.

Some empirical findings make the alleged links between school social relations and children’s cognitive, or working memory development, plausible. The first set of evidence comes from studies on human stress. For example, children suffering from post-traumatic stress disorder (PTSD) show differential brain development and lower executive functioning performances compared to normal control children (Beers and De Bellis 2002; De Bellis et al. 2000). Additionally, acute stress leads to increased brain activity in the pre-frontal cortex, supporting the idea that more resources are needed to cope with stressful situations (Porcelli et al. 2008), which may result in lower working memory performance. Among adults, it was found that psycho-social stress resulted in significant working memory impairments (Schoofs et al. 2008). A second set of evidence comes from studies using lab-experimental designs. Such studies found that experimentally induced social exclusion or rejection leads to decreased performance on cognitive tasks that require retrieval and integration of information in adult and adolescent samples (Baumeister et al. 2002) (see also Baumeister et al. 2005). Finally, a third set of studies focused on normative school social experiences. For instance, using a cross-sectional design, children with emotionally secure relationships with their teachers showed more task involvement, persistence and independence than children with less emotionally secure relationships with their teachers (Koomen et al. 2004; Thijs and Koomen 2008). Although the aforementioned outcomes did not always focus on working memory or even children’s cognitive skills, they do make the hypothesized link plausible. In fact, these studies collectively seem to show that the stress originating from the experience of social problems at school may affect working memory development by continued occupation of cognitive resources. Therefore, is seems likely that social stress at school affects the development of working memory, above and beyond possible reverse paths, in which working memory affects the social experiences that children encounter in school.

## Limitation in our Knowledge

Despite the theoretical plausibility of (transactional) developmental links between school social experiences with teachers and peers and children’s working memory development, the available empirical evidence is limited in several ways. First, the reliance on mainly cross-sectional studies prohibits us from drawing any conclusions on the directionality of effects, or about mutual, transactional links (e.g., Beers and De Bellis 2002; De Bellis et al. 2000; Fahie and Symons 2003; McQuade et al. 2013; Thijs and Koomen 2008). Second, previous work has focused on experimentally induced social stressors like cyberball (Williams and Jarvis 2006), or on extreme social stressors, such as PTSD. For instance, the effect size of experimentally induced social experiences like cyberball typically is above 1 standard deviation, implying a large effect (Williams and Jarvis 2006). It is, however, uncertain how, such experimental situations where all conditions, except for those manipulated in the design, are held equal between participants compare to complex daily life experiences. Similarly, it is unrealistic to assume that a poor teacher-child relationship or poor relations with peers have similar impact on children’s development as a traumatic experience resulting in PTSD. Third, previous work omitted assessments of both relationships with teachers and peers and study of these links in the early elementary school period. Both sources of influence need to be considered as interlinks between teacher and peer experiences and child outcomes have been reported during the childhood years (Mercer and DeRosier 2008; Spilt et al. 2014). Moreover, and as an additional limitation, studies addressing the kindergarten to early elementary school period are much needed as the impact of social experiences on children’s developmental outcomes may be especially profound in those early years of elementary school (see Ladd 2006). Lastly, possible gender differences in the association between children’s social relationships and their working memory development need to be addressed. We know that girls generally have more positive and less negative relationships with their teachers (e.g., Howes et al. 2000) and peers (Rubin et al. 2007). However, whether these differences in social experiences have differential effects on working memory development for boys and girls is largely unknown. One study among adult men and women who were exposed to either a socially stressful or a non-stressful social condition found that social stress was associated with increased working memory performance scores for men, but decreased working memory performance scores for women (Schoofs et al. 2013). Knowledge on possible differences in the links between the child’s relations with teachers and peers and working memory development in young children is lacking.

## The Present Study

The objective of this study is to assess whether working memory facilitates the pro-social relational development of children, while simultaneously, such social experiences with teachers and peers facilitate working memory development. These cross-time associations are studied in a sample of 1109 children followed across 2 school years residing in mainstream Dutch elementary schools. We hypothesized that working memory would predict children’s social relationships with both teachers and peers. Specifically, we expected that higher levels of working memory would be associated with the development of more positive and less negative social relations with teachers and peers. Secondly, above and beyond such links from working memory to social relations, we expected social relations with teachers and peers to predict the development of working memory. That is, we expected that positive social relations would be associated with higher working memory development, whereas negative social relations with lower working memory development. With regards to gender differences we did not have a specific a priori hypothesis as the one previous study showing sex differences (Schoofs et al. 2013) used a cross-sectional design, rather that a study of change as we do, and used adults, not children. Therefore, in our study we will explore for gender differences in the associations between variables without proposing a specific hypothesis regarding the direction of effect.

## Method

### Participants

This study used data from a large-scale ongoing longitudinal project aimed at assessing the psychosocial development of young elementary school children. Elementary schools from both an urban and a rural area in the eastern part and central part of the Netherlands were approached and invited to participate. The first 19 elementary schools that accepted the invitation were included in the project in which both kindergarten and first year classes were targeted for participation. In the Netherlands, children start kindergarten on the day of their fourth birthday. Children typically attend kindergarten for 2 years and move to first grade elementary school at around age 6 years. In the Netherlands, children receive a new teacher every school year during the elementary school years, except for during kindergarten where children stay with the same teacher during the 2 years of kindergarten. In this study, 68.8 % of children received a new teacher between school years. Within participating schools, a total of 1330 children from 61 first and second year kindergarten and first grade elementary school classes were targeted for inclusion in the project. Parents were informed about the project and asked for their approval for their child to participate in the study and 93.1 %, *N* = 1238, 50.1 % boys, of the parents consented to their child’s participation in the project. The majority of children, 97.7 %, and their parents, 87.4 % of mothers and 86 % of fathers, were born in The Netherlands. This percentage is somewhat comparable to the Dutch population, 89.3 % was born in the Netherlands (Statistics Netherlands 2014b). 15.3 % of the children were from low socio-economic status (SES) families. This percentage is lower than that of the general Dutch population, 29.5 % (Statistics Netherlands 2014a).

Children were first assessed in the fall of 2011 (T1) and assessed for a second time approximately 6 months later, in the spring of 2012 (T2). One school year later, in the spring of 2013 (T3), children were assessed for a third time. The average amount of time in between T1 and T2 was 6.37 months, *SD* = 1.05 months, and there was on average 11.70 months between T2 and T3, *SD* = 1.21 months. The present study included data from children that participated at least twice in the working memory task (*N* = 1142). Thirty-three children were excluded from further analyses because parental consent was retracted, bringing the total number of children included in this study to 1109 (555 boys, 554 girls). The mean age of children was 5.52 years at T1,*SD* = 1.00, range = 4.00–8.25 years, 6.02 years at T2, *SD* = 1.00, range = 4.09–8.75 years, and 6.98 years at T3, *SD* = 1.01, range = 5.10–9.90 years. Children included in the study, did not differ from excluded children (those that had participated once or whose permission was retracted), with regard to gender distribution, *χ*^2^(1) = 1.99, *p* = .36, or working memory scores at baseline, *F* (1, 781) = .76, *p* = .38. However, children that were excluded were more likely to come from low SES families than included children, 28.6 % versus 14.4 %, *χ*^2^(1) = 9.09, *p* < 0.01.

### Procedure

The medical and ethical committee at the VU University Medical Center approved the procedures used in this study. Children within classrooms were tested individually on an extensive battery of tests that lasted approximately 30 min in the morning, followed by a 30 min session in the afternoon, during regular school days. During lunch breaks and after school hours, teachers were interviewed and completed questionnaires regarding individual pupils in their class. All tests and interviews were administered by trained assistants who were second year (or higher) bachelor or master students of psychology. Children received token rewards (such as a sticker) for their participation throughout the day and received a small gift at the end of the day to thank them for their participation. Teachers received a box of chocolates and a 25-euro voucher for their participation in the project.

### Measures

Working memory was assessed using a visuo-spatial working memory task (de Kieviet et al. 2012; Klingberg et al. 2005; Klingberg et al. 2002b; Nutley et al. 2009). Although only visuospatial working memory was included, this may be a good indication of overall working memory as Kane et al. (2004) found that correlations between verbal and visuospatial working memory tasks were high, sharing 70–85 % of their variance. As we needed to test multiple children in the same space in a school, the visuospatial working memory task, which could be completed on a tablet computer, was deemed the most suitable. A 4-by-4 grid appeared on the screen, yellow dots then appeared one at a time in different locations on the grid. When the dots had disappeared children were asked to recall the position of the dots in reverse order, as this requires working memory activity. Four practice trials were administered to make sure children understood the task. Starting with two dots that had to be remembered, task difficulty increased after four trials to include an extra dot to be remembered up to and including 7 dots. Within the four trials with a similar number of dots to be remembered, two trials were relatively easy and the subsequent two were relatively hard. The latter two trials were more difficult as dots appeared farther away from each other and also made more ‘crossovers’ on the grid than were made during the first two trials. The task was terminated when children could not correctly recall dot-locations for both trials within the relatively easy or relatively hard trials. To obtain maximum spread in the data (and similar to Kessels et al. 2000), total scores were calculated by multiplying the number of correctly repeated trials by the sub-level reached. The task is a developmentally sensitive and valid measure of working memory in young children (Fry and Hale 1996; Klingberg et al. 2002a).

The quality of the teacher-child relationship was assessed using the Young Children’s Appraisals of Teacher Support (Mantzicopoulos and Neuharth-Pritchett 2003; Spilt et al. 2010). In this task children were asked about the amount of Warmth and Conflict they experienced in their relationship with their teachers. To account for children’s limited reading ability, items were printed as statements on individual slips of paper and read out one at a time to children. A total of 11 items were used to assess relationship warmth (e.g., my teacher says nice things about my work), and 10 items tapped into relationship conflict (e.g., my teacher tells me that I am doing something wrong). Warmth and conflict items covered different contexts, including, but not only, the school or work-related context. Other examples of items are My teacher likes my family (warmth) or My teacher tells me to do work that is too hard for me (conflict), or My teacher tells me I am going to get in trouble a lot (conflict). A binary answer scheme was used. Similar to Spilt et al. (2010), children deposited items in a miniature toy trashcan if they disagreed and in a miniature toy safe if they agreed with items. Mean scores were calculated to represent relationship warmth and conflict within the teacher-child relationship. Although the average amount of conflict experienced by children was relatively low, only 14.3, 17.9 and 18.9 % of children, for T1-T3 respectively, reported to experience no conflict at all. This indicates that the majority of children reported experiencing conflict with their teacher to a certain extent. Cronbach’s alphas ranged from .56 to .59 for warmth and .61–.71 for conflict which correspond to values reported by Mantzicopoulos and Neuharth-Pritchett (2003), who validated the Y-CATS. We additionally fitted confirmatory factor models to confirm that the two-factor model had good fit to the data. Fit indices were good, range *CFI* = .92–.94; *TLI* = .91–.94; *RMSEA* = 0.03, with the correlation between the latent factor warmth and conflict ranging between−.38 and−.48, *p* < 0.001.

Children’s likeability was assessed with peer nominations. Children were asked to nominate all children in the class whom they ‘liked’. Children could nominate everyone in their class, but were not allowed to nominate themselves. As reading levels of children were limited, photographs of the children were used for the nominations. Children were photographed against identical backgrounds and asked to look straight into the camera without smiling. During the test day children were asked to point to the photos of children they liked, which were recorded by the test assistant. Likeability was defined as the total number of times a child was nominated as liked by a peer, divided by the class size minus one (as children could not nominate themselves). Total scores could therefore range from 0 (not liked by any peers) to 1 (liked by all peers). Assessing likeability using peer nominations is a valid and reliable procedure (Wasik 1987).

Dyadic friendedness was assessed by asking children to nominate their best friends. Children could nominate everyone in their class. Friendships were classified as dyadic if their friends also nominated them in return. Total scores of dyadic friendships were divided by the number of children in the class minus one (children could not nominate themselves as best friend). Total scores could therefore range from 0 (no dyadic best friends) to 1 (dyadic best friends with every child in the class).

### Control Variables

Behavior problems were assessed using the conduct problems scale of the Strengths and Difficulties Questionnaire filled out by the children’s teachers (SDQ-T, Goodman and Scott 1999). The conduct problems scale consists of 5 items scored on a 5-point Likert scale (1 = *not at all applicable*, 5 = *very applicable*). Cronbach’s alphas ranged from .81 to .84 over the three assessments.

Household SES was reported by the child’s parents. Parents were asked to report on mothers’ and fathers’ current or most recent job. Job descriptions were classified according to the working population classifications scheme (Statistics Netherlands 2010). The highest SES score of the two parents was taken as children’s SES. SES was dummy coded, with low SES defined as being unemployed or holding a lower or elementary job or less (for example being a cleaner).

The age of the child was added as an additional control variable to account for age differences between children attending kindergarten or first grade elementary school.

### Statistical Analyses

To assess the developmental links between working memory and social experiences, we first assessed descriptive statistics of our study variables. We used repeated measures ANOVAs to assess for gender differences and assessed correlations between our study variables. All these preliminary analyses were conducted using SPSS Statistics version 21. Subsequently, a series of nested cross-lagged models (Jöreskog 1970; van Lier and Koot 2010) were fitted. First we fitted a baseline model that included auto-regressive paths and all within-time correlations between our study variables. Additionally, this baseline model included all cross-lagged paths between study variables that were not part of our hypotheses (e.g., between teacher and peer relational measures). Cross-lagged paths between social relational factors and working memory were not included at this stage. To test our first hypothesis that working memory predicted the development of social relations with teachers and peers over and above the paths already included in the baseline model, regression paths were added from working memory to social relational factors. Lastly, to assess the hypothesis that social experiences with teachers and peers predicted the development of working memory, regression paths between social relational factors and working memory were added. To assess for gender differences in the strength of the associations, a multiple-group model was used.

All models were fitted in Mplus version 6.0 (Muthén and Muthén 1998–2010). As children were either in kindergarten or first grade elementary school at the start of the study we regressed all paths on children’s age to control for mean level-differences. We additionally controlled for family SES. To control for the possible influence of behavioral problems, we included teacher-reported conduct problems as a time-varying covariate in the model. Standard errors of path estimates were adjusted to account for clustering of data within schools using a sandwich estimator (Williams 2000). Comparison between tested models was done using the Satorra-Bentler chi-square difference test (Sattora 2000). Model fit was determined using the Comparative Fit Index (CFI) and Tucker Lewis Index (TLI), and the Standardized Root Mean Square Residual (SRMR). For the CFI and TLI values of .95 and higher indicate acceptable fit (Bentler and Bonett 1980), values of .08 on the SRMR and lower indicate acceptable fit (Marsh et al. 2004).

## Results

### Descriptive Statistics

Table 1 depicts means and standard deviations of all study variables for boys and girls separately. Controlling for multiple assessments using repeated measures ANOVA, we found that working memory levels did not differ between boys and girls, but gender-differences were found for teacher-child relationship conflict and warmth and peer-likeability. Dyadic friendedness did not differ between boys and girls. More specifically, we found that boys experienced on average higher levels of conflict and lower levels of warmth in their relationship with their teacher, and were on average liked less by their peers compared to girls. The time x gender interactions were not significant, indicating that the development over time for each of the constructs is similar for boys and girls. Table 2 depicts correlations between all study variables. Working memory correlated significantly and negatively with teacher-child relationship conflict, irrespective of time of assessment. Working memory and teacher-child relationship warmth correlated significantly and positively at most time points. Working memory additionally correlated significantly and positively with likeability and dyadic friendedness, although correlations were not significant at all time-points. Despite that the magnitude of some of the correlations was modest, significant correlations in the expected directions were found between the study variables. We therefore moved on to testing our hypotheses in the longitudinal cross-lagged framework.

**Table 1:** Means and standard deviations of working memory and social relational factors for boys and girls separately

	Boys			Girls			Test		
	*M*	*SD*	Range	*M*	*SD*	Range	Time	Time*Gender	Gender
WM T1	18.90	14.52	0.00–66.00	17.14	13.23	0.00–71.50	210.11***	.91	.22
WM T2	22.17	15.72	0.00–91.00	21.47	15.25	0.00–97.50			
WM T3	29.99	17.56	0.00–97.50	30.52	17.80	0.00–104.00			
TC conflict T1	.29	.21	0.00–1.00	.24	.19	0.00–0.90	35.47***	.36	28.67***
TC conflict T2	.29	.22	−1.00	.23	.21	0.00–1.00			
TC conflict T3	.24	.19	−.90	.20	.18	0.00–0.90			
TC warmth T1	.84	.15	0.36–1.00	.87	.14	0.36–1.00	3.96*	2.26	19.47***
TC warmth T2	.82	.17	0.09–1.00	.87	.14	0.27–1.00			
TC warmth T3	.85	.15	0.00–1.00	.88	.13	0.00–1.00			
Likeability T1	.27	.19	0.00–1.00	.31	.19	0.00–1.00	62.57***	2.92	11.45**
Likeability T2	.24	.15	0.00–1.00	.26	.15	0.00–0.88			
Likeability T3	.30	.18	0.00–1.00	.35	.19	0.00–1.00			
Friends T1	.13	.12	0.00–0.67	.15	.12	0.00–1.00	11.01***	1.17	.40
Friends T2	.11	0.09	0.00–0.60	.11	0.09	0.00–0.50			
Friends T3	.13	.12	0.00–1.00	.13	.11	0.00–1.00			

Test statistics come from repeated measures ANOVA and represent F-values

*WM* working memory, *TC* teacher-child

* *p* < 0.05, ** *p* < 0.01, *** *p* < 0.001

**Table 2:** Correlations between working memory and social relational factors

Variables	1	2	3	4	5	6	7	8	9	10	11	12	13	14
1. WM T1	-													
2. WM T2	.55*	-												
3. WM T3	.44*	.59*	-											
4. TC conflict T1	−.15*	−.16*	−.16*	-										
5. TC conflict T2	−.15*	−.26*	−.26*	.39*	-									
6. TC conflict T3	−.16*	−.24*	−.23*	.25*	.37*	-								
7. TC warmth T1	−0.02	0.08*	0.03	−0.05	−0.07	−.13*	-							
8. TC warmth T2	0.06	.16*	.12*	−.13*	−.17*	−.15*	.27*	-						
9. TC warmth T3	0.09*	.15*	.11*	−0.09*	−0.09*	−.21*	.21*	.18*	-					
10. Likeability T1	0.09*	.15*	0.09*	−0.07*	−.10*	−0.08*	.10*	0.08*	0.06	-				
11. Likeability T2	.15*	.26*	.19*	−.10*	−.15*	−.14*	.16*	.11*	.10*	.46*	-			
12. Likeability T3	.14*	.18*	.14*	−0.07	−.17*	−.17*	0.05	0.07*	.13*	.31*	.40*	-		
13. Friends T1	.10*	0.09*	0.05	−0.08*	−0.01	−0.06	0.08*	0.07	0.02	.58*	.36*	.38*	-	
14. Friends T2	0.08*	.16*	.17*	−0.03	−.10*	−0.09*	.12*	.12*	.08*	.40*	.55*	.30*	.43*	-
15. Friends T3	.10*	0.04	0.04	−0.05	−0.10*	−0.06	.10*	0.07*	0.08*	.16*	.20*	.57*	.30*	.20*

*WM* Working memory, *TC* Teacher-child relationship, *T1* fall 2011, *T2* = spring 2012, *T3* spring 2013

* *p* < 0.05

### Model Fitting

**Fig. 1:**
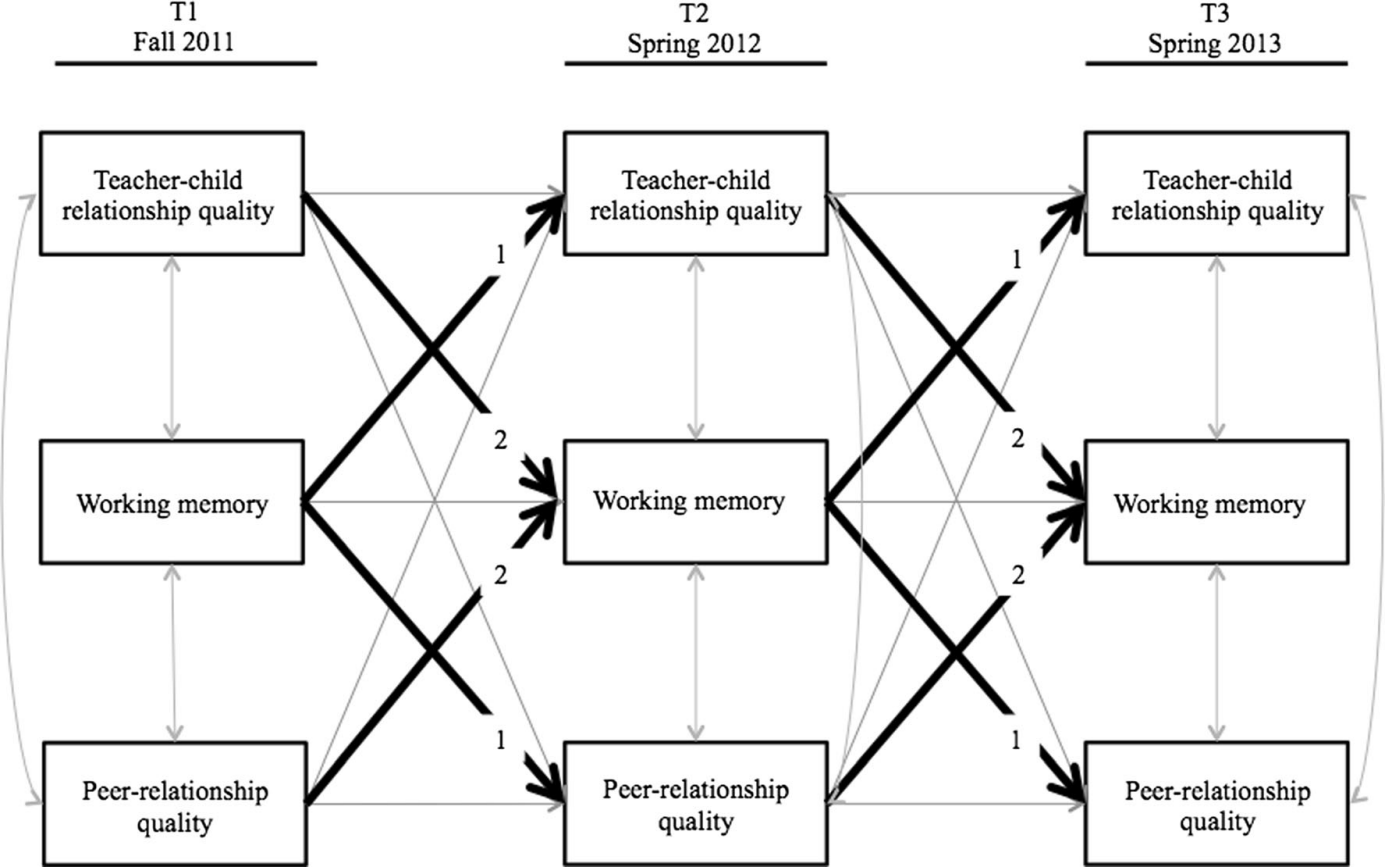
Graphical representation of our hierarchical model fitting strategy. After fitting the base model, regression paths from working memory to social relational factors were added (numbered with 1), followed by paths from social relational factors to working memory (numbered with 2)

Figure 1 depicts our model fitting strategy and Table 3 depicts fit indices of the different models fitted.

**Table 3:** Fit statistics and model comparisons for nested models

	Model Fit					Model Comparison			
Model	*Χ* ^2^	df	CFI	TLI	SRMR	Comparison	ΔΧ^2^	*Δdf*	*p*
1. Baseline	265.47	127	.96	.94	0.06				
2.WM to social relations	227.17	119	.97	.95	0.05	2 versus 1	39.46	8	<0.001
3. Social relation to WM	206.35	111	.97	.96	0.05	3 versus 2	21.93	8	0.01
Gender differences									
4. Gender-unconstrained	296.13	222	.98	.97	0.05				
5. Gender-constrained	356.93	301	.99	.98	0.06	5 versus 4	61.13	79	.93

Model comparison ΔΧ^2^ statistics are based on the Satorra-Bentler chi-square difference (Sattorra and Bentler 2001)

To assess whether working memory predicted social experiences above and beyond the stability and possible interlinks between social experiences with teachers and peers, we started with a baseline mode. In this model, we included auto-regressive paths and within-time correlations between all study variables. Additionally, this baseline model included cross-lagged paths between all study variables except for cross-lagged paths between social relational factors and working memory. This model had an acceptable fit to the data (see Table 3 for fit indices).

We then tested our first hypothesis (see Fig. 1), namely whether social relational factors were predicted by working memory over and above the paths included in the baseline model. Allowing for the paths from working memory to social relational factors resulted in a significant improvement of model fit, ∆*χ*^2^ (8) = 39.46, *p* < 0.001; see Table 3.

To test our second hypothesis (see Fig. 1), namely whether social relational factors predicted the development of working memory above and beyond reverse paths, regression paths between social relational factors and working memory were added. This again resulted in a significant improvement of model fit∆*χ*^2^ (8) = 21.93, *p* = 0.01, and resulted in a good fit to the data, *CFI* = .97, *TLI* = .96, *SRMR* = 0.047.

### Social Experiences and Working Memory Development

**Fig. 2:**
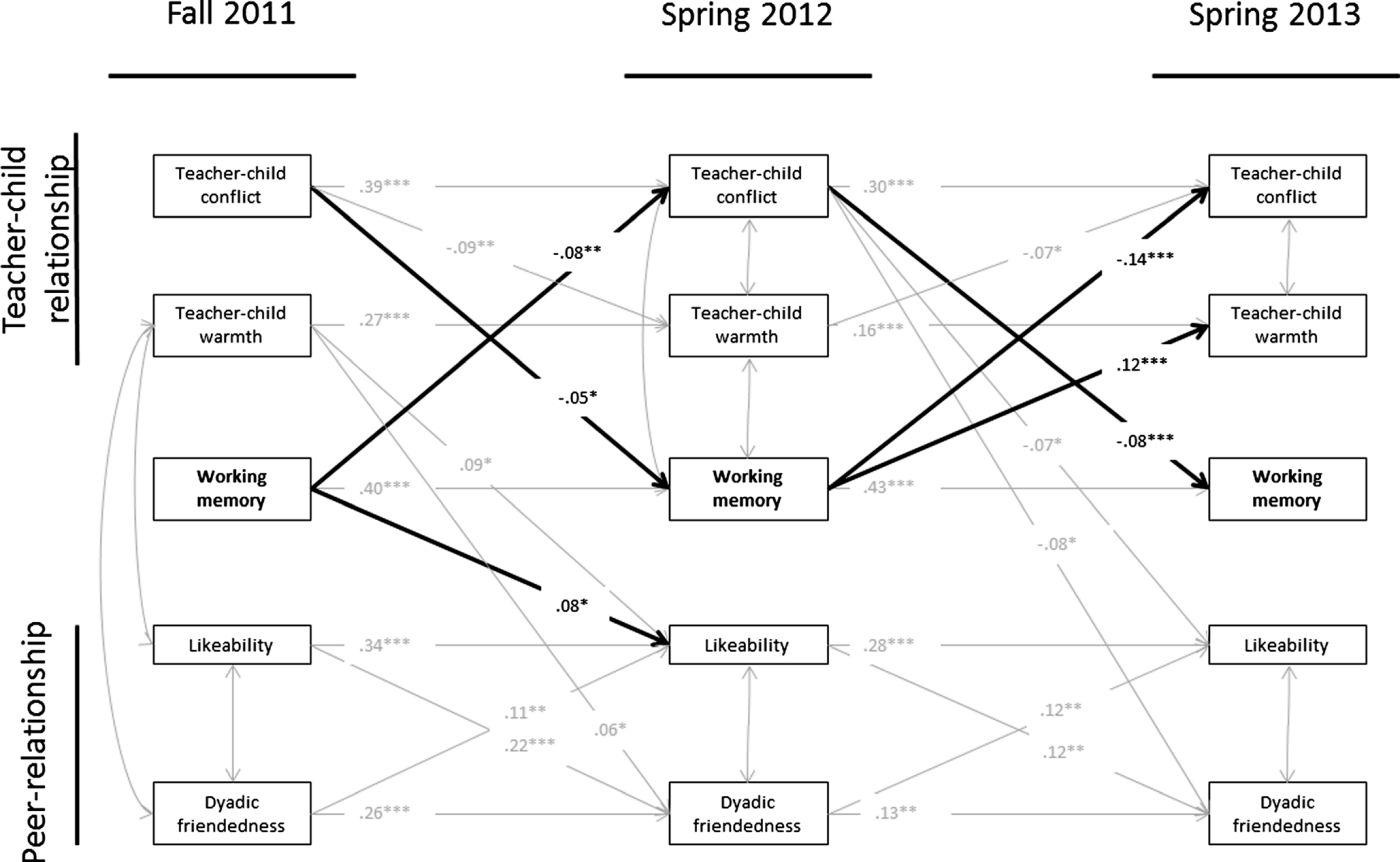
Cross-lagged model between social relational factors and working memory. Only significant paths have been depicted, *p* < 0.05. Path coefficients are only displayed for paths relevant to the hypotheses. *CFI* = .97, *TLI* = .96, *SRMR* = 0.05. *** *p* < 0.001, ** *p* < 0.01, * *p* < 0.05

Standardized results of the final model are depicted in Fig. 2. In support of our first hypothesis results show that working memory was negatively linked over time to teacher-child relationship conflict within the same school year (T1 to T2) as well as between school years (T2 to T3). Working memory was positively associated with later teacher-child relationship warmth between school years, but not within the school year. Controlling for whether or not children received a new teacher between school years did not affect our model results. With regards to peer-relationships, working memory associated negatively with later child likeability within the school year but not between school years.

We also found support for our second hypothesis; conflictual teacher-child relationships over time was significantly and negatively associated with later working memory development within and between school years.

### Gender Differences

To assess whether significant path estimates between working memory and social relationship factors were similar for boys and girls a multiple group model was fitted. First, we fitted a model in which all paths were freely estimated across gender. Consequently, we compared this model to a model in which all paths were constrained to be equal across gender. Differences in model fit were assessed using the Satorra-Bentler chi-square difference test (Sattorra and Bentler 2001; Sattora 2000). Results indicated no significant differences between genders, ∆*χ*^2^ (79) = 61.13, *p* = .93.

## Discussion

The goal of this study was to examine the developmental links between young children’s working memory and their social development. As anticipated, working memory performance was associated over time with children’s social relations with their teachers and peers. In addition to this, we found support for social experiences being associated with children’s working memory development. Higher levels of conflict within the teacher-child relationship was related to lower working memory development. These results therefore suggest that working memory performance is related to children’s emerging social relations with teachers and peers, but that working memory itself is in turn associated with earlierclassroom social experiences.

With regards to our first hypothesis, our finding that working memory performance links to children’s social relationships with teachers and peers is in line with previous research (Fahie and Symons 2003; McQuade et al. 2013). Although previous research assessed verbal working memory instead of visuo-spatial working memory, verbal and visuospatial working memory have been found to share 70–85 % of the variance (Kane et al. 2004). Moreover, other studies used a different visuospatial working memory task than what was used in our study. Nevertheless, Kane et al. (2004) also report high correlations between different tasks measuring visuospatial working memory. Therefore, our findings are in line with previous studies. However, this study extends previous results by showing these results using a conservative and complex longitudinal design, among children from the general population. Although the magnitude of effect sizes in our study are small, the effects are in the expected directions and are measured repeatedly over time within a normative sample. Note that we used a very conservative longitudinal design in which we assessed predictive links of changes in study variables where all variables were assessed in parallel. Therefore, the significant paths may yield important information as they reflect influences of real life effects on working memory beyond possible non-included compensating factors in children’s lives, across an important developmental span of kindergarten/early elementary school year. Using such a design we found that over time lower working memory performance was associated with increases in conflictual social experiences with teachers suggesting that working memory is important for developing satisfying relationships in the classroom. Children with higher working memory performance may be better at learning from their teacher’s feedback and may be more flexible in adjusting their behavior appropriately, hence reducing conflict with their teacher. Moreover, because we had data across school years, our results showed that children with poorer working memory scores continue to develop poor relationships even when a new teacher emerged in the classroom after the transition to a new school year.

With regards to effects of working memory on positive aspects of classroom relations our results showed that working memory performance linked to later teacher-child relationship warmth between school years, but not within the school year. This somewhat contrasts the pattern of links between working memory and teacher-child conflict, which was found both within and between school years. It has been argued that negative experiences are more influential on children’s psychosocial development than positive experiences (Baumeister et al. 2001). Extending this, it may be that negative relationship characteristics are also more susceptible to influences by children than positive relationship characteristics. The association between working memory and teacher warmth, a positive aspect of the teacher-child relationship, suggests that higher working memory was associated with more subsequent warmth by teachers. Our results suggest that children with good working memories swiftly develop a positive relationship with the teacher, and once established, this remains stable. Note that the first, cross-time link reflected the continuity of the relationship with the same teacher within the first study year. As a new teacher emerged in our second study year, the link between working memory and student-teacher warmth across school years may actually reflect the emergence of the new positive relationship. The single link found between working memory and likeability from T1 to T2 may reflect that working memory still associates with likeability in the early years of formal schooling. However, as classroom composition with regards to children remains stable between school years, it may well be that once established, the predictor loses its influence on the likeability score.

In line with our second hypothesis, we also found support for the social environment being associated with working memory development. Both within, as well as between school years teacher-child relationship conflict was associated with later working memory performance. This finding is in line with theoretical, as well as empirical evidence (Baumeister et al. 1996, 2002, 2005; Davies et al. 2008). Our finding that conflict within the teacher-child relationship negatively linked to working memory development, supports both theoretical frameworks, and extends previous research in two important ways. First, it extends the previous findings that were mostly based on experimentally induced social experiences (Williams and Jarvis 2006) or extreme stress (Beers and De Bellis 2002; De Bellis et al. 2000) to children’s every day social relationship experiences in school. Second, it shows that such every day social experiences may affect working memory development during the formative years of elementary school. Generally accepted as developing over time (Gathercole et al. 2004; Riggs et al. 2006), children’s working memory may not only develop naturally and thereby concurrently influence their social experiences, but may in fact also be affected by this social development during this time. It is especially this latter finding that is important as children’s social experiences in the classroom are associated with their previous working memory scores, while simultaneously their social experiences in the classroom are associated with their working memory development. Our results thus suggest bidirectional, or transactional links between classroom social processes and working memory development in children. The fact that working memory is malleable is in line with recent studies showing that working memory can be trained by continuously practicing working memory rather than changing strategies for keeping information in working memory (e.g., Klingberg et al. 2002a, b). Our results suggest that working memory development is hindered when children experience conflictual relationships with their teacher. Theoretically it may be that as children first aim to restore their relationship, resources cannot be spent on cognitive development. When children experience their relationships not improving, they finally give up, further eliminating opportunities to practice their working memory (see also Williams 2009).

With regards to gender differences in these effects and despite mean level differences in teacher-child relationship quality and peer likeability between boys and girls we found no evidence suggesting sex-differences in the developmental links between working memory and social relational factors. Therefore, the reported effects between working memory and social relational factors apply similarly to boys and girls. This finding seems contradictory to Schoofs et al. (2013), who found that social stress affected working memory differently for men and women. However, our study design was very different to theirs. In contrast to our longitudinal sample of young children, Schoofs and colleagues assessed the association between social stress and working memory cross-sectionally, using an adult sample. To our knowledge, there have been no other studies assessing gender differences in the association between working memory and social relational factors.

We have to add to our discussion that our effects were particularly evident for the teacher-child relationship. We found no support for links between peer social experiences and working memory development. Moreover, we found that the teacher-child relational impact on working memory was limited to teacher-child conflict; experienced warmth from the teacher did not link to working memory development. One possible explanation could be that especially negative aspects of social relations are associated with working memory development, with positive social experiences linking to a lesser extent. In an extensive review, Baumeister et al. (2001) similarly concluded that bad experiences bear more power than good experiences across different domains, including the social domain. This may also explain the differences in impact by teachers versus peers. For peers, we did not have relational factors indicating conflictual relationships or aversive social experiences. That is, although being liked by few children or having few friendships may be troublesome, the impact of such an experience on a child is likely different than that of being disliked by children or being overly excluded from social interaction.

### Limitations & Implications

This study has several limitations. First, our sample consisted mainly of white, Dutch children with relatively few children of low SES families. Although our sample was comparable to the Dutch population it would have been interesting to assess whether our results extend specifically to more diverse populations, including children from minority backgrounds. Second, as stated, our study did not include a measure of negative social aspects of peer-experiences, such as peer rejection. Future studies should test whether such negative aspects of peer experiences link more consistently over time to working memory development, as was the case with negative aspects of the teacher-child relationship. Thirdly, the teacher-child relationship quality scales that we included in our study had low alphas. Although this is a limitation, which may have reduced the associations between variables, the task has been validated for this age-group with the original validation study showing similar alphas (Mantzicopoulos and Neuharth-Pritchett 2003). Despite the low alphas, the results were robust and in the expected directions. Additionally, our confirmatory factor model showed that the two-factor model had a good fit to the data, suggesting children could clearly distinguish between items tapping into warmth from items tapping into conflict. Therefore, although the estimates were low, the low alphas did not seem to invalidate the results of this study.

Despite these potential limitations, our findings have implications for future research as well as for practice. Using a conservative longitudinal design in kindergarten and an early elementary school sample, our study confirms the role of working memory in children’s social development. Future studies predicting social problems should attempt to include an executive function measure or working memory in their models. More importantly, our results showed that there is likely a transactional relationship between working memory development and social experiences in the classroom. Part of the development of working memory depends upon the relationship that children have with their teachers, which in part is evoked by the child’s working memory capacity. Future studies, both on the development of cognitive skills and social experiences during kindergarten and early elementary school, should thus take into account this bidirectional influence.

Our results have implications for prevention. Working memory is not only important for social development, but also for further cognitive and academic development. The findings of transactional effects between young children’s social development and working memory suggest that prevention should be most effective when fostering social classroom relationships in young children, while simultaneously training children’s working memory. Although interventions aimed at each of these components exist, they are generally not integrated. For instance, programs like The Good Behavior Game (Barrish et al. 1969) are aimed at fostering teacher-child relationships or prosocial relationships with peers, while programs like Cogmed (Åkerlund et al. 2013; Klingberg et al. 2005) are aimed at training children’s working memory. Both programs are applicable to the school context, but are not integrated. Our results on bidirectional relationships urge combining programs aimed at improving both working memory as well as social relations, to optimize the effectivity of both intervention programs.
